# Oleanolic Acid Suppresses Migration and Invasion of Malignant Glioma Cells by Inactivating MAPK/ERK Signaling Pathway

**DOI:** 10.1371/journal.pone.0072079

**Published:** 2013-08-21

**Authors:** Guocai Guo, Weicheng Yao, Quanqin Zhang, Yongli Bo

**Affiliations:** 1 Department of Neurosurgery, The Affiliated Hospital of Medical College, Qingdao University, Qingdao, China; 2 Department of Internal Medicine, The Affiliated Hospital of Medical College, Qingdao University, Qingdao, China; National Cancer Center, Japan

## Abstract

Mitogen-activated protein kinases/Extracellular signal-regulated kinase (MAPK/ERK) pathway is essential for migration and invasion of malignant glioma. It is efficient to inhibit migration and invasion of glioma cells by targeting this pathway. Oleanolic acid (OA) has been well demonstrated to suppress survival, growth and angiogenesis of glioma cells. However, it is still unknown if OA affects the migration and invasion of glioma cells. We utilized U-87 MG glioma cell lines and primary glioma cells from patients to study the effect of OA on migration and invasion of glioma cells with multidisciplinary approaches. In this study, we found that OA significantly decreased the ability of glioma cells to migrate and invade. Epithelial-mesenchymal transition (EMT) of glioma cells was also suppressed by OA treatment. Furthermore, MAPK/ERK pathway was greatly inhibited in glioma cells under OA treatment. MAPK/ERK reactivation induced by a recombinant lentiviral vector, Lv-MEK, was able to rescue the inhibitory effect of OA on migration and invasion of glioma cells. Taken together, we provided evidences that OA was a MAPK/ERK pathway-targeting anti-tumor agent. Although the concentrations we used exceeded its physiological level, OA may be used to prevent migration and invasion of glioma cells by developing its derivatives with enhanced bioactivity.

## Introduction

Malignant glioma is the most common primary brain tumor with high migration and invasion [1]. Chemotherapy is one of the most feasible therapeutic modalities for the patients who suffered from glioma invasion. However, chemotherapy is always not effective enough in glioma treatment, primarily because most of the existing drugs are not designed for targeting the pathways critical for migration and invasion of glioma cells. Therefore, it is required to develop specific pathway targeting agents to suppress glioma migration and invasion [2].

Accumulated evidences showed that glioma cells depend on MAPK/ERK signalling pathways to undergo migration and invasion [3]–[5]. Suppression of MAPK/ERK signalling activity compromises migration and invasion ability of glioma cells [6]–[9]. Therefore, MAPK/ERK pathway was believed to be an effective therapeutic target in glioma anti-invasion treatment.

As a potent anti-tumor agent, oleanolic acid (OA) suppressed many malignant phenotypes of glioma cells [10]–[14]. Intriguingly, OA and its derivatives has no cytotoxicity to normal human cells [15], [16]. These inhibitory effects of OA are involved with its suppression of some specific intracellular signaling pathways, such as STAT3, JNK, Akt and NF-kappaB signaling pathways [10], [12], [14]. However, the anti-migration activity of OA on glioma cells has not been investigated yet.

In this study, we intended to examine if OA could suppress migration and invasion of glioma cells. Our results showed that OA inhibited these properties of malignant glioma cells via targeting MAPK/ERK pathways.

## Materials and Methods

### Compounds and Cell Line Culture

OA were purchased from Sigma-Aldrich (Code Number: O5504). Human glioblastoma cell lines, U-87 MG and U-251 MG cells were purchased from American Type Culture Collection (Manassas, VA) and were cultured using DMEM supplemented with 10% of fetal bovine serum (FBS), 4 mM glutamine, 100 units/ml penicillin, and 100 µg/ml streptomycin in a 5% CO_2_ and humidified atmosphere at 37°C.

### Primary Glioma Culture/Ethics Statement

We employed primary cultures derived from malignant glioma in this study. For primary glioma culture, fresh cancerous tissue was obtained with written informed consent from all patients according to protocols approved by Ethical Review Board in the Affiliated Hospital of Medical College of Qingdao University (Qingdao, China). All patients underwent surgical resection of primary glioma at Department of Neurological Surgery, The Affiliated Hospital of Medical College of Qingdao University (Qingdao, China). Glioma tissues were cut into small pieces. The single cell suspension was obtained by mechanical manipulation. The primary cultures were established initially in DMEM supplemented with 15% FBS and maintained in DMEM supplemented with 10% FBS.

### Migration and Invasion Assay

For migration assay, 5×10^4^ cells were resuspend in 200mL of serum-free media and seeded onto the upper chamber of 24-well hanging cell culture insert (Millipore) fitted with polyethylene terephthalate (8.0 µm pore size). 900 ml DMEM media with 20% FBS was added to lower chamber of each well. After 48 h, cells were fixed by 4% paraformaldehyde and then dyed with crystal violet.

For invasion assay, the upper chamber of 24-well hanging cell culture insert was pre-coated with Matrigel (BD Biosciences, Sparks,MD, USA). The following procedures were the same as that of migration assay.

### Wound Healing Migration Assay

U-87 MG and primary glioma cells (GT-1^#^) were planted in a six-well plate at the concentration of 4×10^5^ cells per well. 8 h later, cells were cultured in starvation media for overnight 37°C incubation. A scratch wound was made in each well of the 6-well plate using a pipette tip, followed by being washed with starvation media to remove any loosely held cells. OA was added to the starvation media at the concentration of 5, 10 and 25 µg/mL (10, 20 and 50 µM). Images were captured at the indicated time-points.

### MTT Assay

Cells were cultured in 96-well plates at 1–1.5×10^4^ cells per well. Overnight, OA of the indicated concentrations were administrated on cells. 48 h later, 50 µL of 3-(4, 5-dimethylthiazol-2-yl)-2, 5-diphenyltetrazolium bromide (MTT; 1 µg/mL) was added to cell media. 4 h later, MTT was discarded and 150 µL of DMSO were loaded in each well. The spectrophotometric absorbance of the samples was measured with Microplate Reader Model 550 (Bio-Rad Laboratories, Japan) at 570 nm with a reference wavelength of 655 nm. The percentage of cell survival was calculated using the following formula: cell survival = absorbance value of infected cells/absorbance value of uninfected control cells. Eight reduplicate wells were measured at each concentration and every experiment was performed at least three times.

### Apoptosis Detection

3.5×10^5^ cells were cultured in each well of 6-well plates. Overnight, the cells were treated with OA of indicated concentrations. 48 h later, the cells were washed and then stained with Annexin V-PI Apoptosis Detection Kit (Biovision, CA) according to the manufacturer’s instructions. The percentage of apoptotic cells was analyzed through FACS.

### Cell Cycle Analysis

3.5×10^5^ cells were cultured in each well of 6-well plates. Overnight, the cells were treated with OA of indicated concentrations. 48 h later, cell cycle analysis was done by the evaluation of DNA content by PI staining. The cells were prepared as a single cell suspension of 1–2×10^6^ cells/mL in PBS. After the cells were fixed with cold 70% ethanol for 2 h, the cells was washed twice with PBS and then stained with Propidium Iodide (PI) at the final concentration of 50 µg/mL with RNase at 20 µg/mL in PBS. The treated cells were then evaluated by FACS analysis.

### Quantitative PCR (qPCR)

Total RNA was extracted from glioma cells with Trizol solution (Sigma-Aldrich, MO), followed by being reversely transcribed into cDNAs via Rever Tra Ace qPCR RT Kit (Toyobo, Japan) according to the manufacturer’s instructions. qPCR was performed using SYBR premix Ex Taq (TaKaRa) on CFX96™ Real-Time PCR Detection System (Bio-Rad Laboratories, CA) supplied with analytical software. The primers used for qPCR are described in **Table 1**.

**Table 1:** Primer sequences used for detection of EMT-associated gene expression in qPCR assay.

Gene	Primer sequences
CDH1(E-Cadherin)	F: 5′-TGCCCAGAAAATGAAAAAGG-3′
	R: 5′-GTGTATGTGGCAATGCGTTC-3′
CDH2(N-Cadherin)	F: 5′-ACAGTGGCCACCTACAAAGG-3′
	R: 5′-CCGAGATGGGGTTGATAATG-3′
VIM(Vimentin)	F: 5′-GAGAACTTTGCCGTTGAAGC-3′
	R: 5′-GCTTCCTGTAGGTGGCAATC-3′
TWIST1	F: 5′-GTTAGGGTTCGGGGGCGTTGTT-3′
	R: 5′-CCGTCGCCTTCCTCCGACGAA-3′

### Immunoblotting Assay

Proteins were harvested with M-PER® Mammalian Protein Extraction Reagent (Thermo Scientific, IL), separated using polyacrylamide gel electrophoresis and transferred onto 0.45 µm nitrocellulose membranes. The membranes were blocked with 5% fat-free dry milk in PBS and incubated with corresponding primary antibody overnight. The used antibody include anti-E-cadherin (1∶400, BD), anti-N-cadherin (1∶400, Santa Cruz), anti-vimentin (1∶500, Abcam), anti-Twist1 (1∶500, Abcam), anti-MEK1 (1∶1000, Santa Cruz), anti-phosphorylated MEK1 (1∶1000, Santa Cruz), anti-ERK (1∶800, Santa Cruz), anti-phosphorylated ERK (1∶1000, Santa Cruz) and anti-GAPDH (1∶1000, Cell signaling). After that, the membrane was incubated with corresponding secondary antibodies and visualized with SuperSignal West Dura Extended Duration Substrate (Thermo Scientific, IL).

### Luciferase Reporter Assay

Cells were plated in a 96-well plate at 5×10^3^ cells per well. After 24 h, cells were transfected with MAPK/ERK pathway-specific Cignal SRE Reporter (luc) Kit (CCS-010L) following the manufacturer’s instructions. After 24 h, OA was added to cell cultures. After 6 h, luciferase expression level in these transfected cells were assessed using Bright-Glo luciferase assay kit (Promega, Madison, WI) following the instructions. Cells without OA treatment were used as standard. Relative luciferase activities in these cells were shown as the ratio between their luciferase activity and standard, both of which were normalized with background.

### Lentiviral Vectors

A lentiviral vector carrying full-length *MEK1* genes (Lv-MEK) and a control vector (Lv-GFP) were generously provided by Dr. Liu (Qingdao University, Qingdao, China). Transcription of *MEK1* and *GFP* genes on this plasmid were driven by human elongation factor 1a (EF1a) promoter. The titers of these two recombinant lentiviruses were determined following the previous procedures [17].

### Statistical Analysis

All values were reported as means ± SD, and compared at a given time point by unpaired, two-tailed student t test. Data were considered to be statistically significant when P<0.05 (*) and P<0.01 (**).

## Results

### OA Affected Migration and Invasion of Glioma Cells

**Figure 1:**
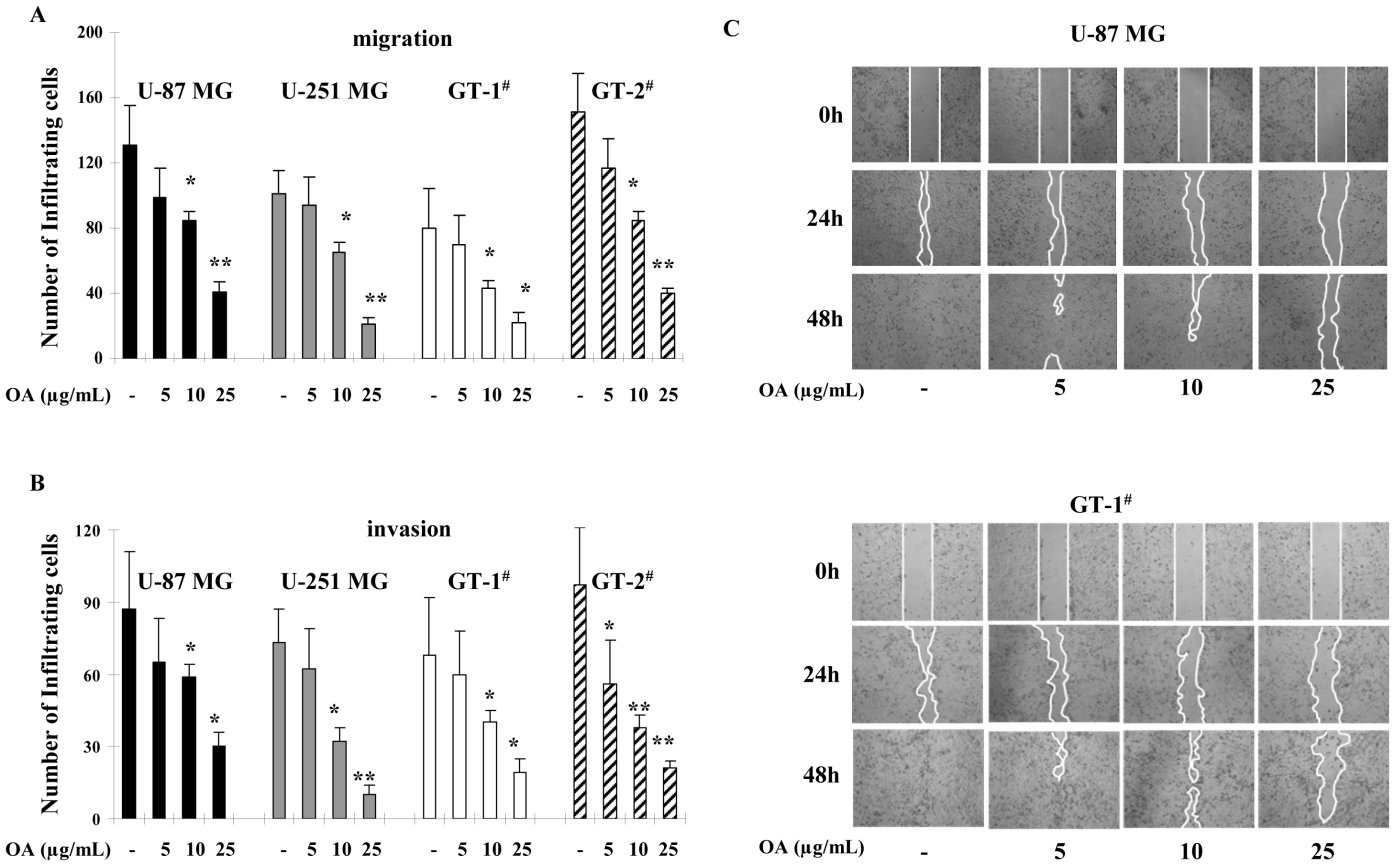
Inhibitory effect of OA on migration and invasion of glioma cells. (**A**) 5, 10 or 25 µg/mL (10, 20 and 50 µM) of OA was added to the culture of U-87 MG, U-251 MG and two primary glioma cells (GT-1^#^ and GT-2^#^). 48 h later, the number of cells that passed through the membranes was counted in five separate fields (100×). The average values were shown with ± SD. (**B**) The experiments were performed in the same conditions except for the use of Matrigel-precoated membranes instead of uncoated ones. (**C**) 4×10^5^ indicated glioma cells were seeded in 6-well plates. Overnight, a linear area of attached cells was removed by a pipette tip when indicated concentrations of OA were administrated. The cells were photographed at the same time and after 24 h and 48 h (100×). The line confined the area not covered with cells.

We employed transwell assays to investigate if migration and invasion of glioma cells were affected by OA. Significantly, fewer U-87 MG cells were found to infiltrate the membranes with and without Matrigel under 48 h treatment of OA at the concentrations of 5, 10 and 25 µg/mL (10, 20 and 50 µM), compared with untreated groups **(** **Fig. 1a and 1b** **)**. Interestingly, OA also inhibited migratory and invasive properties of U-87 MG cells even at a low dose (10 µg/mL) **(** **Fig. 1a and 1b** **)**.

Also, we investigated if OA showed a similar effect on migration and invasion of glioma cells other than U-87 MG. Consistently, we found that fewer U-251 MG and primary glioma cells went through the uncoated and Matrigel-coated membranes when exposed with 5, 10 and 25 µg/mL (10, 20 and 50 µM) of OA **(** **Fig. 1a and 1b** **)**.

Furthermore, we applied wound healing migration assay to confirm the inhibitory activity of OA on migration of U-87 MG and primary glioma cells (GT-1^#^). After 24 h and 48 h treatment of 5, 10 and 25 µg/mL (50 µM) of OA, migration of the tested glioma cells were all compromised **(** **Fig. 1c** **)**. In addition, OA displayed no significant cytotoxicity, apoptosis-stimulating activity or cell cycle arrest effect at the above concentrations **(Fig. S1).**

The above data demonstrated that, within a dose range where apoptotic and cell cycle arrest event did not occurred, OA was able to suppress the migration and invasion of glioma cells in a dose- and time-dependent manner.

### OA Inhibited Epithelial-mesenchymal Transition (EMT) of Glioma Cells

**Figure 2:**
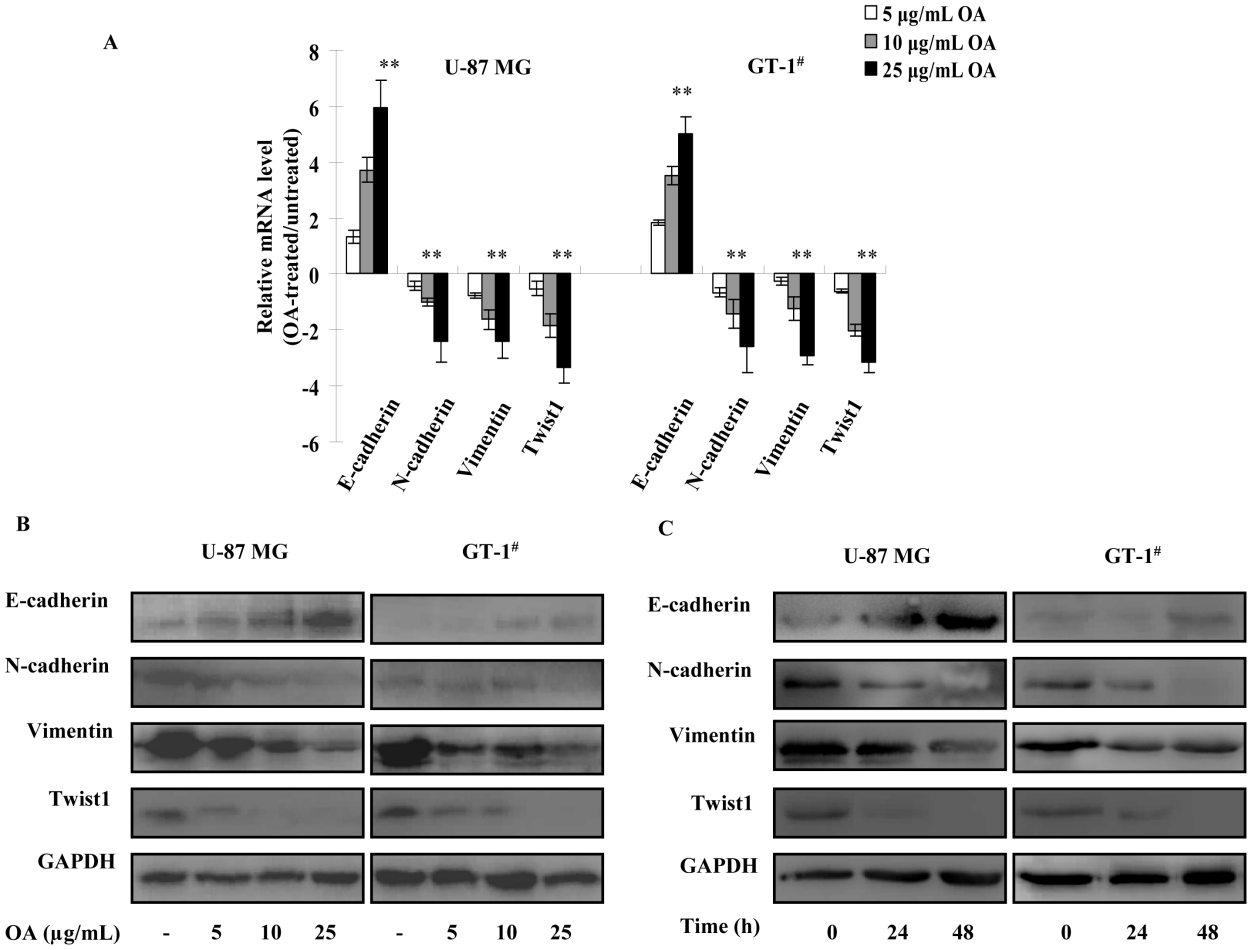
OA treatment suppressed EMT process of glioma cells. (**A**) 5, 10 or 25 µg/mL (10, 20 and 50 µM) of OA was added to the culture of U-87 MG and GT-1^#^ cells. 48 h later, transcripts of E-cadherin, N-cadherin, Vimentin and Twist1 were extracted and quantified. The experiments were performed for three times. *GAPDH* was selected as endogenous control. Their relative expression levels in OA-treated cells to untreated ones were shown as log_2_ (mean ± SD). (**B**) Under 48 h treatment of 5, 10 or 25 µg/mL (10, 20 and 50 µM) of OA, E-cadherin, N-cadherin, Vimentin and Twist1 proteins were also determined and GAPDH was selected as endogenous control. (**C**) After 24 h and 48 h treatment of OA, the above proteins were assessed in U-87 MG and GT-1^#^ cells treated by 25 µg/mL (50 µM) of OA.

Regarding that EMT is closely associated with migration and invasion of cancer cells, we subsequently investigated the effect of OA on EMT processes of glioma cells by examining the changes in E-cadherin, N-cadherin, Vimentin and Twist1 expression. qPCR and immunoblotting assays both demonstrated that the expression of epithelial cell marker, E-cadherin, was elevated in U-87 MG and GT-1^#^ cells under 48 h treatment of 5, 10 and 25 µg/mL (50 µM) of OA in a dose-dependent fashion. Meanwhile, expression levels of mesenchymal cell markers N-cadherin and Vimentin, as well as the key EMT inducer Twist1, were all decreased in OA-treated cells under the same conditions **(** **Fig. 2a and 2b** **)**. Furthermore, the changes in the expression level of these EMT-related markers displayed a time-dependent manner under OA treatment **(** **Fig. 2c** **)**.

Collectively, OA inhibited the EMT process in glioma cells and induced glioma cells to gain epithelial properties in a dose- and time-dependent manner.

### OA Suppressed the Activation of MAPK/ERK Pathway in Glioma Cells

**Figure 3:**
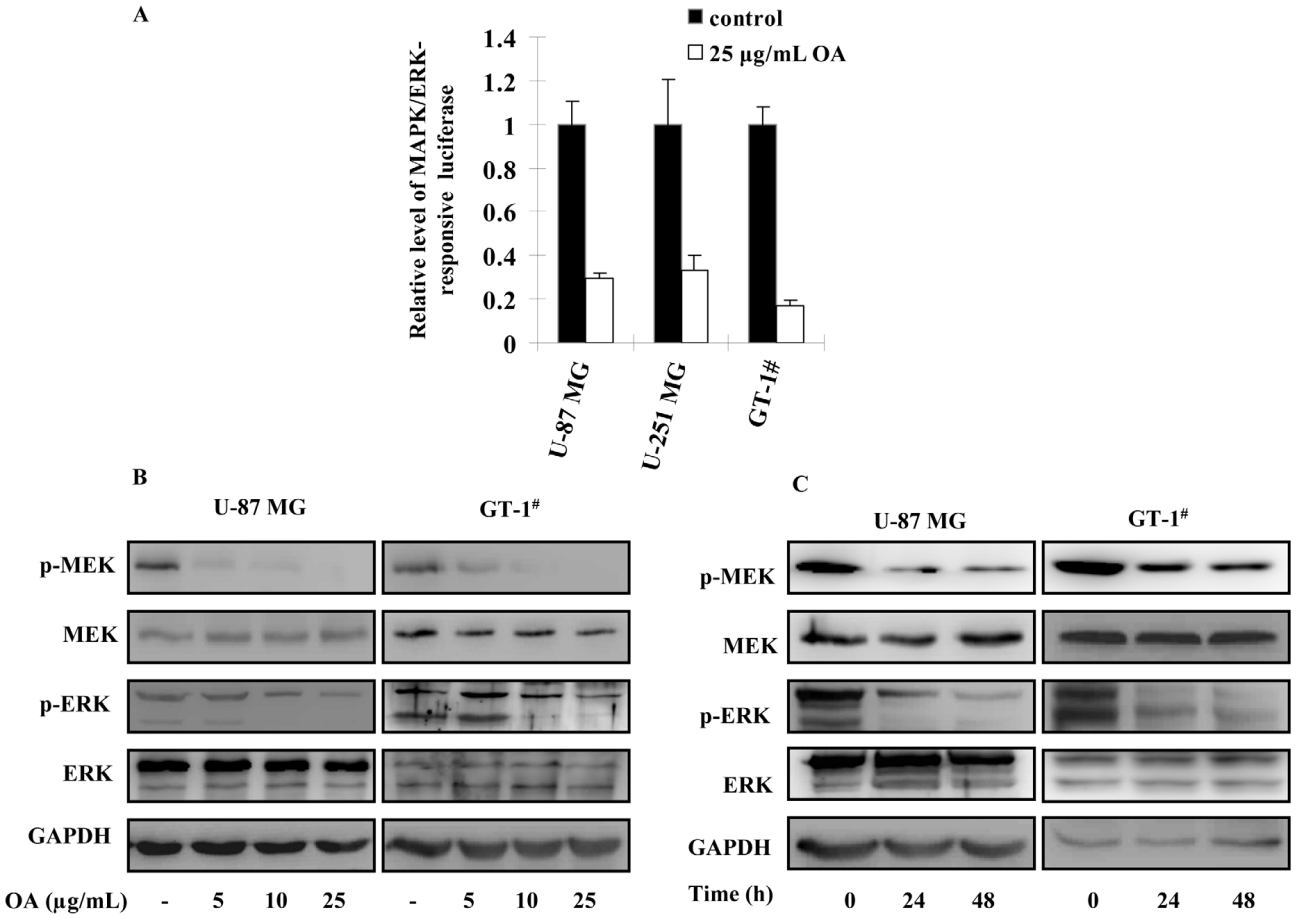
MAPK/ERK signaling activation was suppressed in OA-treated glioma cells. (**A**) 1×10^4^ U-87 MG, U-251 MG and GT-1^#^ were planted in 96-well plates. Cignal SRE Reporter was applied on the cells to detect the activation of MAPK/ERK signaling according to the instructions. 24 h later, 25 µg/mL (50 µM) of OA was added to the cultures. 6 h later, the luciferase activity was quantified and the level of Firefly luciferase was normalized by Renilla luciferase. The luciferase activity in the untreated cells was selected as standards. This experiment was repeated for three times and the values were shown as mean ± SD. (**B**) 48 h after 5, 10 or 25 µg/mL (10, 20 and 50 µM) of OA treatment, MEK and ERK proteins as well as their phosphorylated forms were also determined in U-87 MGand GT-1^#^ cells and GAPDH was selected as endogenous control. (**C**) The above proteins were also detected in U-87 MG and GT-1^#^ cells treated by 25 µg/mL (50 µM) of OA at the indicated timepoints.

Given MAPK/ERK pathway plays an essential role of the migration and invasion of glioma cells, we used luciferase reporter and immunoblotting assays to determine if OA affected its activation. The results showed that activation of MAPK/ERK pathways was suppressed by greater than 70% in glioma cells when 25 µg/mL (50 µM) of OA was added **(** **Fig. 3a** **)**. Immunoblotting assays also confirmed that phosphorylation level of MEK (also MAPKK, mitogen activated protein kinase kinase) and ERK (extracellular regulated protein kinases) was reduced by 48 h treatment of 5, 10 and 25 µg/mL (50 µM) of OA in U-87 MG and GT-1^#^ cells in a dose-dependent fashion, suggesting MAPK/ERK signaling pathway was inactivated under OA stimulation **(** **Fig. 3b** **)**. Also, the suppressing activity of OA on MAPK/ERK signaling pathway showed a time-dependent manner **(** **Fig 3c** **)**.

### Lentivirus-based MAPK/ERK Pathway Activation Abolished the Effect of OA on EMT of Glioma Cells

**Figure 4:**
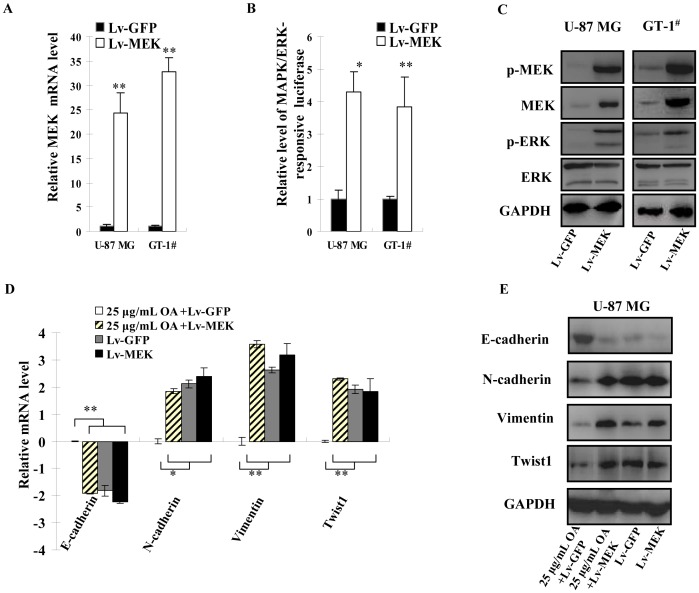
Lentivirus-based MAPK/ERK pathway activation abolished the effect of OA on EMT of glioma cells. (**A**) 10 MOI of Lv-MEK or Lv-GFP was added to the cultures of U-87 MG and GT-1^#^ as well as 25 µg/mL (50 µM) of OA. 48 h later, total RNA was extracted from the cells and expression level of MEK mRNA was determined by qPCR. The experiments were performed for three times. *GAPDH* was selected as endogenous control. Their relative expression levels in OA-treated cells to untreated ones were shown as mean ± SD. (**B**) Under the same conditions, MAPK/ERK-responsive luciferase plasmids were transfected into U-87 MG and GT-1^#^ cells and Firefly luciferase activity was normalized by that of Renilla luciferase. The luciferase activity in untreated cells was selected as standards. These experiments were repeated for three times and the values were shown as mean ± SD. (**C**) Immunoblotting assay was perfermed to detect MEK and ERK proteins as well as their phosphorylated forms were also determined in 10 MOI of Lv-MEK or Lv-GFP-infected U-87 MG and GT-1^#^ cells under 25 µg/mL (50 µM) of OA treatment and GAPDH was selected as endogenous control. (**D**) 10 MOI of Lv-MEK or Lv-GFP was used to infect U-87 MG cells with or without 25 µg/mL (50 µM) of OA. 48 h later, transcripts of E-cadherin, N-cadherin, Vimentin and Twist1 were quantified. The experiments were performed for three times. *GAPDH* was selected as endogenous control. Their relative expression levels in the tested cells to ones treated by 25 µg/mL (50 µM) of OA and 10 MOI of Lv-GFP were shown as log_2_ (mean ± SD). (**E**) E-cadherin, N-cadherin, Vimentin and Twist1 proteins were also determined in the cells treated simultaneously by lentivirus and 25 µg/mL (50 µM) of OA and GAPDH was selected as endogenous control.

To confirm the importance of MAPK/ERK pathway inactivation for OA to inhibit migration and invasion potentials of glioma cells, we overexpressed MEK1 protein by infecting a recombinant lentivirus. qPCR, luciferase reporter and Immunoblotting assays confirmed that lentivirus infection (10 MOI of Lv-MEK) resulted in elevated expression of *MEK1* gene **(** **Fig. 4a and 4c** **)** and reactivated MAPK/ERK pathway in U-87 MG and GT-1^#^ cells under OA treatment **(** **Fig. 4b and 4c** **)**.

According to the data from immunoblotting and qPCR assays, E-cadherin expression was decreased in MEK-overexpressing OA (25 µg/mL, 50 µM)-treated U-87 MG glioma cells while there was no difference in its expression between MEK-overexpressing and control cells without OA treatments **(** **Fig. 4d and 4e** **)**. N-cadherin, Vimentin and Twist1 levels were increased in MEK-overexpressing U-87 MG cells under OA treatment **(** **Fig. 4d and 4e** **)**. These results showed that MEK restoration was able to abolish the inhibitory activity of OA on EMT process in U-87 MG glioma cells.

### Reactivation of MAPK/ERK Pathway Rescued The Inhibitory Effect of OA on Migration and Invasion of Glioma Cells

**Figure 5:**
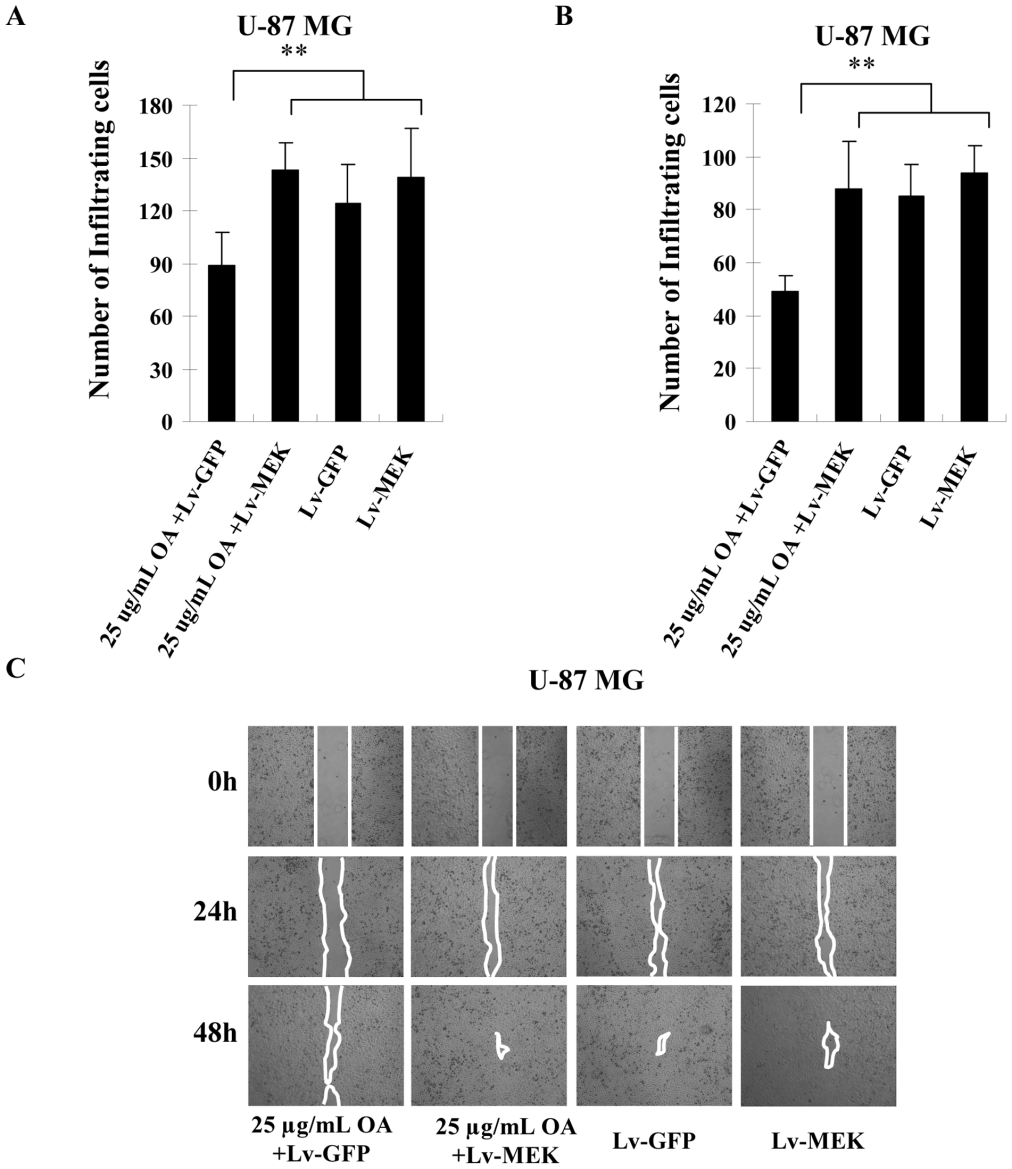
Reactivating MAPK/ERK signaling rescued anti-migration and anti-invasion capacity of OA on glioma cells. (**A**) 48 h after the treatment of indicated lentiviruses or/and OA, the number of U-87 MG cells that passed through the membranes was counted in five separate fields (100×). The average values were shown with ± SD. (**B**) The experiments were performed in the same conditions except for the use of Matrigel-precoated membranes instead of uncoated ones. (**C**) 4×10^5^ U87 MG glioma cells were seeded in 6-well plates. Overnight, a linear area of attached cells was removed by a pipette tip when 25 µg/mL (50 µM) of OA or PBS was administrated. The cells were photographed at the same time and after 24 h and 48 h (×100). The line confines the area not covered with cells.

MEK overexpression also increased the number of OA (25 µg/mL, 50 µM)-treated glioma cells that went through the membranes with and without Matrigel **(** **Fig. 5a and 5b** **)**. In contrast, MEK overexpression did not alter the migration and invasion ability of U87 MG cells when OA was not applied **(** **Fig. 5a and 5b** **)**. Wound healing migration assay also confirmed the influence of MEK overexpression on migration ability of U-87 MG cells. Both after 24 h and 48 h treatment of 25 µg/mL (50 µM) OA, MEK restoration compromised its anti-motility activity on U-87 MG cells **(** **Fig. 5c** **)**.

These data proved that inactivated MAPK/ERK signaling is required for the inhibitory effect of OA on glioma migration and invasion.

## Discussion

OA also possessed anti-metastasis capacity on some types of cancers. A group found that OA treatment inhibited lung metastasis of B16F10 melanoma cells in mice model [18]. Osteosarcoma cells were also shown to have reduced incidences of lung metastasis when exposed with an OA derivative [19]. In this study, we originally provided evidences that OA could inhibit migration and invasion of glioma cells. We selected 5, 10 and 25 µg/mL OA to perform the subsequent experiments, because higher dose of OA showed cytotoxicity to glioma cells. Our data demonstrated that OA was able to suppress the migration and invasion of glioma cells even at a low dose.

Although vimentin protein has been found to be disorganized in the astrocytoma cells treated by high dose of OA, it has not been determined whether its expression level was also affected in the same conditions [12]. Our data showed that Vimentin expression was suppressed by OA, even at a low dose. Expression levels of two other mesenchymal related proteins, N-cadherin and Twist1, consistently declined in the OA-treated glioma cells. These results indicated that glioma cells underwent mesenchymal-epithelial transition (MET) in the presence of OA, which may account for its inhibitory function on migration and invasion of glioma cells.

MAPK/ERK pathway played important roles in multiple biological behaviors of glioma cells. Its activation is required for the expression of VEGF, an important angiogenic factor for glioma, in two glioma cell lines under normoxic conditions [20]. MAPK/ERK activation also participated in the protein kinase C-eta isoform-induced proliferation of glioblastoma cells [21]. Recently, activation of this signaling pathway was reported in CD133^+^ glioblastoma stem cells, suggesting its possible role in the maintenance of cancer stem cell stemness [22]. New evidences also indicated that PTPIP51 accelerated the formation of human glioblastoma via activating MAPK/ERK pathway [23]. Importantly, MAPK/ERK activation is closely associated with EMT initiation and progression in glioma cells. The disruption of EMT in medulloblastoma cells caused by downregulation of uPA/uPAR has been shown to be mediated by MAPK/ERK signaling [24]. Our results revealed that MAPK/ERK signaling was potently suppressed by OA. MAPK/ERK signaling pathway was reported to be involved with both TGF-β and non-TGF-β-induced EMT process [25], [26]. Furthermore, we used lentiviral vectors to overexpress MEK1 protein to reactivate MAPK/ERK pathway in OA-treated glioma cells, finding that the gene transfer significantly rescued OA inhibition of glioma migration and invasion. Our results showed that MAPK/ERK signaling suppression is required for OA’s inhibitory effect on migration and invasion of glioma cells.

Although our data showed that OA reduced the migration and invasion of glioma cells *in vitro*, the dose of OA we used in this study was significantly higher than its physiological level. To address this issue, OA derivatives that have an enhanced bioactivity can be developed. In fact, some OA derivatives have been synthesized for an increased anti-tumor activity [27]. These OA derivatives should be investigated for their anti-migration activity in further studies.

Taken together, we designed and performed experiments to prove that OA suppressed the ability of glioma cells to migrate and invade through inactivating MAPK/ERK signaling pathway. Our data indicated that OA is a promising agent for glioma therapy and may be applied for clinical treatment.

## Supporting Information

Figure S1 **Inhibitory effect of OA on survival and growth of glioma cells. (A)** U-87 MG cells were treated with indicated doses of OA. After 48 h, MTT assay was performed to determine cell viabilities. The bars showed means of the relative values normalized by absorptive values of untreated U-87 MG cells from three independent experiments with SD. **(B)** The same cells were treated by OA of different concentrations. After 48 h, apoptotic rates were evaluated by FACS analysis on Annexin V expression. The bars showed means of data from three independent experiments with SD. **(C)** U-87 MG cells were treated by OA of different concentrations. After 48 h, cell cycle analysis was done by PI staining. Percentages of cells at G0/G1, S and G2/M phases were expressed as bars. The bars showed means of data from three independent experiments.
(TIF)
