# Supplementary figures and images for: Oleanolic Acid Suppresses Migration and Invasion of Malignant Glioma Cells by Inactivating MAPK/ERK Signaling Pathway
Source: PLoS One. 2013 Aug 21;8(8):e72079. doi: 10.1371/journal.pone.0072079 (PMC3749117; doi:10.1371/journal.pone.0072079)

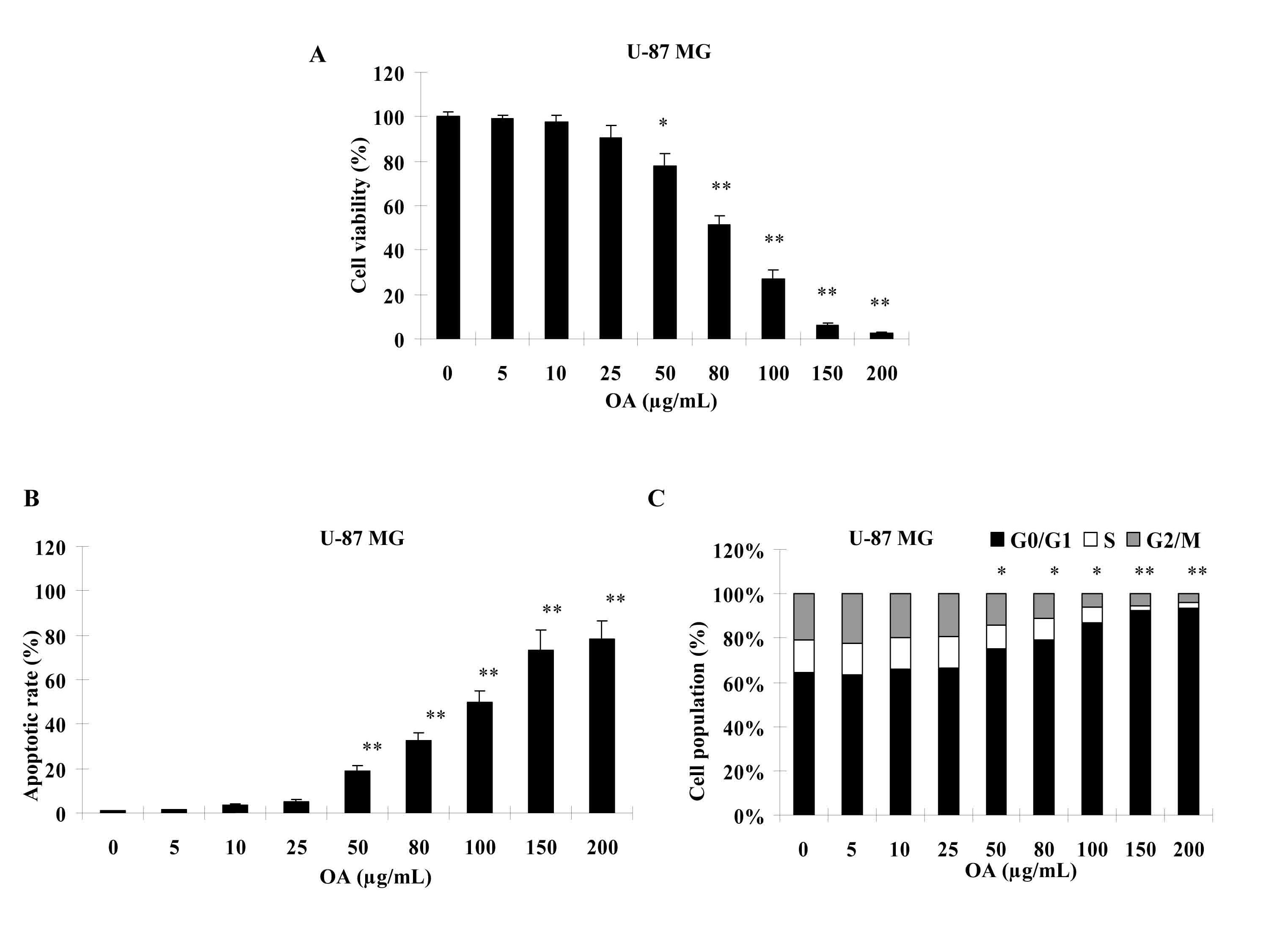

Supplement: Figure S1 — Inhibitory effect of OA on survival and growth of glioma cells. (A) U-87 MG cells were treated with indicated doses of OA. After 48 h, MTT assay was performed to determine cell viabilities. The bars showed means of the relative values normalized by absorptive values of untreated U-87 MG cells from three independent experiments with SD. (B) The same cells were treated by OA of different concentrations. After 48 h, apoptotic rates were evaluated by FACS analysis on Annexin V expression. The bars showed means of data from three independent experiments with SD. (C) U-87 MG cells were treated by OA of different concentrations. After 48 h, cell cycle analysis was done by PI staining. Percentages of cells at G0/G1, S and G2/M phases were expressed as bars. The bars showed means of data from three independent experiments. (TIF) [file pone.0072079.s001.tif]
